# Digital natives of the labor market: Generation Z as future leaders and their perspectives on leadership

**DOI:** 10.3389/fpsyg.2024.1378982

**Published:** 2024-05-31

**Authors:** Betül Yılmaz, Elvin Dinler Kısaçtutan, Seçil Gürün Karatepe

**Affiliations:** ^1^Department of Labor Economics and Industrial Relations, Faculty of Economics, Marmara University, Istanbul, Türkiye; ^2^Department of Labor Economics and Industrial Relations, Faculty of Economics and Administrative Sciences, Trakya University, Edirne, Türkiye; ^3^Department of Business Administration, Faculty of Economics and Administrative Sciences, Istanbul Arel University, Istanbul, Türkiye

**Keywords:** Generation Z, Generation Z leaders, digital natives, leadership, future leaders

## Abstract

**Introduction:**

Today’s labor market is comprised of different generations and in the near future, the majority of it will consist of Generation Z. In this respect, it is of great importance to know the perspective of Generation Z, which will take its place in leadership positions in organizations, on leadership and what kind of leader they will be. The present study probes to investigate how Generation Z will become a leader and their perspective on leadership. In this regard, the study aims to offer suggestions and guidance to the literature and organizations by providing information on what kind of leaders they will be as well as knowing the characteristics of Generation Z leaders.

**Methods:**

In the study conducted Generation Z, employed in the IT sector, semi-structured interviews were held with 13 participants. While analyzing the data obtained from the interviews, the MAXQDA 2020 program was utilized and the thematic analysis method was applied.

**Results:**

In our findings about how Generation Z will be leaders in the future, two themes were identified, which include intra-organizational communication and working conditions. In the light of findings on the leadership of Generation Z, the sub-themes of being supportive, empathetic, egoless, managing people well and managing with love and respect were reached on the theme of intra-organizational communication. On the other hand, on the theme of working conditions, sub-themes such as providing training, offering payment according to output, preferring flexible working hours, being friendly, assigning appropriate work to the employee, being fun and not giving importance to gender were identified.

**Discussion:**

The results revealed that Generation Z prefers managing their team with a people-oriented approach when they take leadership positions. From their perspective, the leader should understand and value his employees. Leaders who know the expectations of Generation Z will contribute to their organizations. In addition, organizations should improve their leadership skills by providing leadership training for Generation Z, who will be leaders in the future. In this way, they will make investment both in their leaders and their organizations.

## 1 Introduction

In today’s global labor markets, employees involve different generations. Within these generational differences, the majority of the workforce consists of Baby Boomers (19%), Generation X (35.5%), and Generation Y (39.4%) (John Hopkins University, 2023). Although these generations form the majority in labor market, it is expected that Generation Z with its more than two billion representatives in the world population will form 30% of global labor force by 2050 (Chomątowska et al., 2022). In the near future, the labor market will be mostly made up of the representatives of Generation Y and Z and Generation Z will begin to assume important positions in this market (Mărginean, 2021; Lazar et al., 2023). In Türkiye, on the other hand, Baby Boomers constitute 6.2% of the labor force, Generation X 25.3%, Generation Y 40% and Generation Z 28.4% (TURKSTAT, 2024). This issue has become very important as Generation Z’s transition to leadership positions will occur in the short term due to their rapid entry into the labor market. It has become necessary to focus especially on the transformations in workplaces and leadership styles. Nevertheless, in the present literature, there is not enough information about the leadership styles and team management of Generation Z (Benítez-Márquez et al., 2022).

One of the best ways to understand what kind of leaders Generation Z, who are considered to be digital natives in the digitalizing world, will be in the future and their perspective on leadership is to focus on IT sector employees. This study was supported by qualitative research to fill the gap in the literature by focusing on Generation Z, who works in the IT sector and aims to become leaders. In studies on Generation Z and leadership, it has been observed that the focus is on the leadership expectations of Generation Z, but there are no studies on how they will be leaders and how they will manage their teams. In this respect, the research questions were formed so as to fill the existing gap in the literature on the leadership styles and team management of Generation Z and to be a source of information that will shape leadership practices in the workplace. While designing the research questions, the focus was on how leaders who design their working lives in the future would be leaders and how they would manage their teams. Therefore, the research questions below form the basis for the present study;

1. What kind of leaders will Generation Z be?
2. How will Generation Z manage their teams when they become leaders?

## 2 Literature review

In studies upon the topic of generation, the common consensus is that the members of Generation Z are accepted as individuals who were born in the second half of the 1990s and grew up in the 2000s (Magano et al., 2020) even though the starting year of Generation Z varies in the literature (Zemke et al., 2000; Williams et al., 2010; Andrea et al., 2016; Bejtkovsky, 2016; Lanier, 2017; Agarwal and Vaghela, 2018; Grow and Yang, 2018; Gaidhani et al., 2019; Chillakuri, 2020). Generations are shaped according to the culture and history they exist, and in order to understand the characteristics of generations, it is necessary to look at the history in which they are shaped (Coomes, 2004) because the generation refers to subcultures which create a common value among people with the widespread influence of the social, economic, political and cultural developments and social environment experienced in the pre-adult period (De Cooman and Dries, 2012).

Although the starting date of Generation Z varies, they are referred to as the first global generation in the world. Generation Z shares the same culture globally, prefers similar foods, fashions and places and this globalization is also reflected in the language they use (Törőcsik et al., 2014). Generation Z, also referred to as digital natives, was born between 1995 and 2010 (Andrea et al., 2016) and witnessed a time when the information age, the internet and digital globalization were on the rise; thus, they have been using the tools of the digital age for most of their lives and they spend their time using these tools (Prensky, 2001; Kapil and Roy, 2014). The motto of Generation Z, which is also expressed in different ways such as “Netgeneration,” “iGeneration,” “Connection-generation,” “Digital-Generation,” “Responsibility-Generation” (Csobanka, 2016), is “Never without the Internet” (Güler, 2016). This generation’s addiction to high technology has become a part of their identity (Singh and Dangmei, 2016). Due to the fact that they spend most of their time in the digital environment, their characteristics are shaped similar to the virtual environment (Ozkan and Solmaz, 2017). They see both the virtual world and the real world as complementary to each other and can switch easily between the two (Dolot, 2018).

The distinctive features of Generation Z involve high self-confidence, knowing what they want, high awareness, ability to express themselves well, wanting to be in control and high sensitivity to social events, independence, being introvert, dissatisfaction, being free-rider, making friends on social media, being weak in social relations, being pragmatic, enterprising and humanist. Moreover, they are tolerant and understanding; their interests are quite high, and they have the ability to perform multiple tasks at the same time (Kütükçü, 2015; Lanier, 2017; Arar and Öneren, 2018; Said et al., 2020; Yılmaz, 2020; Bulut and Maraba, 2021; Duger, 2021; Kılınç and Varol, 2021; Sakdiyakorn et al., 2021).

Individuals of this generation together with their different characteristic structure compared to the previous generations, make these differences felt in their working lives. Generation Z’s expectations from working life, personal satisfaction and goals are different from previous generations. What motivates them to choose the organizations they work for is not only profit but also the desire for a space where they can achieve their personal development (Tidhar, 2022). In addition to the education they get before entering working life, professional training and development that they get after working life are also crucial for them (Dhopade, 2016).

According to the results of the research conducted by EY (2016) on Generation Z, they prefer organizations which provide learning and development opportunities in their careers, enable equal payment and promotion opportunities for all employees regardless of differences, provide job security, support work life balance by offering flexibility in terms of when and where to work and possess a diverse environment in the workplace (trying to recruit, retain and encourage people with all differences such as style, gender, race and opinion).

For Generation Z, the social environment is important in terms of entering working life and creating organizational culture. They prefer a workplace which is open to communication, where they are active rather than a hierarchy and where they can accomplish their entrepreneurial skills. Moreover, a friendly workplace, which provides mentoring and development opportunities, is a tool for them to achieve their dreams. Satisfaction at work is very important for them because they can easily leave their jobs if they are not happy (Bridges, 2015; Ozkan and Solmaz, 2015). A permanent career plan at work is not important for them as they do not like routine work and can easily get bored with their job, which means that they can easily change their workplace (Dolot, 2018; Dwidienawati et al., 2021). The results of studies conducted on the expectations and priorities of Generation Z in working life generally reveal similar and consistent findings (Lanier, 2017; Kızıldağ, 2019; Düzgün, 2020; Pekel et al., 2020; Racolţa-Paina and Irini, 2021; Çaşın, 2022; Ngoc et al., 2022; Uğurbulduk, 2022; Vengrouskie et al., 2023; Koç Aslan et al., 2024).

Leadership is an important factor in organizations to keep Generation Z in the workplace and to make them give up their intention to leave (Elçi et al., 2012). A leader’s ability to retain his employees is directly proportional to his attitude and behavior towards his team. According to the World Economic Forum (2014) “Outlook on the Global Agenda 2015 report,” 86% of the experts stated that the world is in the middle of a leadership crisis and that leaders are inadequate in terms of managing the new workforce and are not sufficiently prepared to meet their needs and expectations. In today’s organizations, leaders, managing different generations, need to get ready for understanding their team, knowing their expectations, and having multi-generational management skills (Stuckey, 2016). Leadership is a key factor in achieving success and therefore, with the uncertainties and changes emerging in today’s job market, organizations must invest in their leaders (Tanaka et al., 2024).

The fact that generations have different value judgments and perspectives also changes expectations and perceptions towards leadership (Bako, 2018; Saracel et al., 2023). There are many studies on the leadership style preferences of Generation Z. It is realized that the leadership style expectations at the center of these studies are visionary, coach and democratic, participatory and charismatic leadership styles (Addor, 2011; Seymen, 2017; Schroth, 2019) However, studies have shown that Generation Z individuals desire to work the least with managers who have a bureaucratic leadership style (Maioli, 2017; Düzgün, 2022; Güleç Bekman and Gündüz, 2022). In this regard, since visionary leadership style involves empathy, self-confidence and change initiation competencies related to emotional intelligence, coach type leadership style involves empathy, self-awareness and other development competencies related to emotional intelligence, democratic leadership style involves collaboration, team leadership and communication competencies regarding emotional intelligence, and the participatory leadership style includes the competencies of employees to participate in decision-making processes in their working lives, it can be emphasized that the expectations of generation Z from their leaders are within this framework (Goleman, 2004; Düzgün, 2022). Research shows that Generation Z employees have expectations regarding their freedom, wages, flexible workspaces, use of technology and personal rights, which requires leaders to satisfy Generation Z and provide the necessary motivation in accordance with these expectations (Lidija et al., 2017; Güleç, 2021). In research conducted on the expectations of Generation Z from their leaders, it has been revealed that they have expectations in that the leaders should be fair, act in accordance with the law, be responsible (Bresman and Rao, 2018; İlhan Nas and Doğan, 2020), act equally, be democratic, and be understanding (Dwidienawati et al., 2021; Gabrielova and Buchko, 2021; Saracel et al., 2023) and it is expected that a management approach that does not put pressure on them or cause them to lose their enthusiasm should be adopted and respected (Aydoğdu and Kara Özkan, 2021; Demirbilek and Keser, 2022).

In recent years, studies on Generation Z primarily focus on learning the distinguishing features and characteristic structures of this generation from the previous generations. These studies concentrate on the effects of Generation Z on the business world over time (Lazar et al., 2023). In studies conducted to examine the impact of Gen Z on the workplace and leadership in the future, they are categorized under the headings such as strengths and weaknesses of Generation Z, values and goals in the workplace, Generation Z and the future of leadership worldwide (Goryunova and Jenkins, 2023). Furthermore, studies on leadership and Generation Z generally focus on the expectations of Generation Z from their leaders and their preferred leadership style (Bolat et al., 2018; Klein, 2018; McGaha, 2018; Aguas, 2019; Avcı, 2020; İlhan Nas and Doğan, 2020; Aydoğdu and Kara Özkan, 2021; Dwidienawati et al., 2021; Gabrielova and Buchko, 2021; Demirbilek and Keser, 2022; Düzgün, 2022; İnal and Akdemir, 2022; Zehetner et al., 2022; Molek et al., 2023; Saracel et al., 2023; Savić et al., 2023; Ruiz-Vázquez et al., 2024; Yavuz Aksakal and Ulucan, 2024). The subjects regarding how Generation Z will, who will be the leaders of the future, transfer their knowledge and skills to future generations (de Boer et al., 2021; Benítez-Márquez et al., 2022), what kind of a leader they will be and their perspective on leadership are also an extremely important issue because understanding what kind of leaders Generation Z will be and their view of leadership can predict the future of leadership and also contribute to the sustainability of organizations and their ability to compete in global markets. In this respect, the aim of the present study is to understand what kind of leaders Generation Z will be in the future in the digitalizing world and their perspectives on leadership.

## 3 Materials and methods

### 3.1 Participants

The participants consist of those who represent Generation Z, live in Türkiye, and were born in 1995 and later. Semi-structured interviews were conducted with a total of 13 participants. Eight of those participants were men and five were women. Participants were found through announcements made on social media. The criteria followed when selecting those who responded to the announcement and were included in the study are as follows; (1) Being involved in working voluntarily, (2) Being born between 1995 and 2010 (Generation Z), (3) Being employed in the IT sector, (4) Aiming to be a leader in career plans, and (5) Having at least 1 year of experience. Additionally, purposeful sampling was utilized when selecting participants. The demographic characteristics of the participants, their sectors and their duties are presented in detail in Table 1.

**Table 1:** Demographic characteristics of participants.

**Participant**	**Gender**	**Age**	**Education**	**Years of experience**	**Sector**	**Title**
Participant 1	Male	24	Electronic Engineering	1 year	Software	Test Automation Engineer
Participant 2	Male	25	Computer Engineering	2 years	Software	Test Automation Engineer
Participant 3	Male	23	Computer Programming	6 years	Digital Marketing	Mid-level Manager
Participant 4	Male	24	Management Information Systems	5 years	Automation	Mid-level Manager
Participant 5	Female	26	Management Information Systems	6 years	Software	Project Manager
Participant 6	Male	25	Computer Engineering	3 years	Automotive	Test Automation Engineer
Participant 7	Male	23	Computer Engineering	1 year	Energy	Data Engineer
Participant 8	Female	27	Economy MA	4 years	Software	Business Development Engineer
Participant 9	Male	26	Labor Economics and Industrial Relations	2 years	Software	Project Manager
Participant 10	Female	27	Computer Engineering	3 years	Banking	Software Specialist
Participant 11	Male	23	Computer Engineering	1 years	Software	Artificial Intelligence Engineer
Participant 12	Female	26	Computer Engineering	2 years	Mobile Application	Mobile Application Developer
Participant 13	Female	25	Computer Engineering	3 years	Software	Test Engineer

### 3.2 Data collection

In the study, the data were collected through use of semi-structured interview method. The interviews were held in September–November 2023 during the hours convenient for the employees. Employed IT experts requested all meetings to be held at night and online due to their flexible working hours and workload. There are five participants who clearly stated that face-to-face meetings would be a waste of time. Eight participants stated that they preferred online meeting. For this reason, semi-structured interviews with a total of 13 participants were conducted online via Microsoft Teams and Zoom applications and lasted for 30–45 min. Before starting the interviews, all participants were informed about the purpose of the study and their consent was obtained by stating that the interviews would be recorded. After being informed that their data would be protected and confidentiality would be ensured, the participants were explained that they could give up the interview at any time. The data were stored in a way that could only be accessed by researchers and each participant was given a number to protect their identities. They were requested to have their cameras on during the interview and notes and field diaries were kept about the participants’ gestures, facial expressions, and emotional states. The interview questions are presented in Table 2. The data collection process continued until saturation was achieved. Before starting the field research, approval was obtained from Trakya University Social and Human Sciences Research Ethics Committee (Decision no: 2023.08.18).

**Table 2:** Interview questions.

1	Who is a leader?
2	How would you describe the best leader?
3	What are the characteristics of a good leader?
4	If you were given a leadership opportunity, what kind of a leader would you become?
5	What kind of leader do you see yourself as in the future?
6	What approach will you take when you become a leader?
7	Do you think the leader has a gender? What gender comes to your mind when you think of a leader?
8	How will you set pages when you become a leader?
9	How will you determine working hours when you become a leader?
10	With what tools do you plan to contribute to personal and professional development of your team?

### 3.3 Data analysis

**Figure 1:**
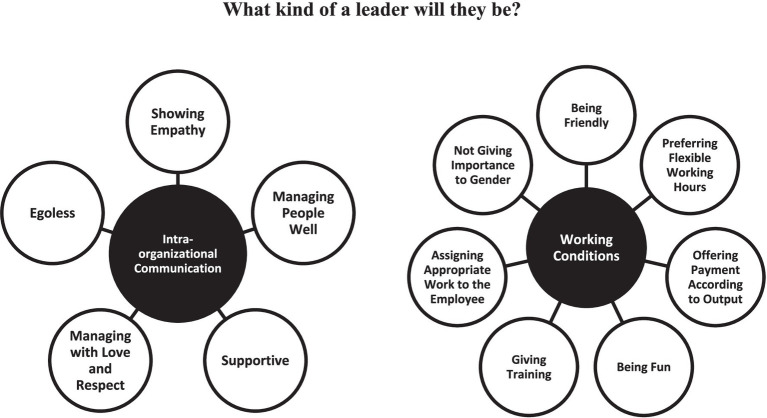
Themes.

Thematic analysis was conducted by the researchers during data analysis process. Thematic analysis is a flexible method, which partially differentiates thematic analysis from other qualitative methods (Terry et al., 2017). Thematic analysis can provide detailed and rich data description (Braun and Clarke, 2006). The reason why thematic analysis was selected as the method in the study is its flexibility. During the data analysis period, after the interviews were transcribed inductively by the first author, the transcripts were read multiple times by all authors and uploaded to the MAXQDA 2020 program by the second author. During the data analysis process, the authors followed the steps suggested by Braun and Clarke (2006). Based on this, the second author reread the entire data set, coded it systematically and looked for themes. In addition, the second author reviewed, narrowed and defined the themes that might emerge. The first author then reviewed the themes and checked the coding. The process of defining and naming the themes continued until a joint decision was made by the first author and the second author by discussing the themes and they took their final form. The final themes are displayed in Figure 1. After the naming process of the themes was finalized, the report was written.

### 3.4 Limitations of the study

There are many limitations in the study. The study was carried out with people who were born, raised, educated and resided in Türkiye. Moreover, all of the participants work in the IT sector. Therefore, the study may not represent all sectors.

## 4 Results

Findings from the interviews were categorized under two main themes, which involve Communication within the organization and Working Conditions. For the theme of Intra-Organizational Communication, five sub-themes were formed, which are supportive, empathetic, egoless, managing people well and managing with love and respect. For the second theme, working conditions, a total of seven sub-themes were determined, which are providing training, offering payment according to output, preferring flexible working hours, being friendly, assigning appropriate job to the employee, being fun and not giving importance to gender. Figure 1 presents the themes and sub-themes.

### 4.1 Theme 1: intra-organizational communication

#### 4.1.1 Supportive

This sub-theme focuses on the significance of supporting employees in the intra-organizational communication of Generation Z leaders. The participants, who emphasize that the support received from the leader is very important in working life and that employees should be guided, point out that the main duty of a leader is to teach and support employees about their job. Future leaders believe that supporting people whom they see as striving for self-improvement and business will have an effect that spreads throughout the organization. Furthermore, they do not see any harm in getting support from their teams in areas they do not know.

*“I try to provide more leadership and support to those who are really trying to pave the way for them. I would try to guide them and direct the more successful people in my team to them so that everyone can reach the same level.”* (Participant 10).

*“Even if there are things I do not know, I will get their support. I will also be supportive, non-judgmental and helpful. These will be my first characteristics.”* (Participant 13).

*“Actually, the leader’s duty is to enable more or less support. Teach your job, support, and develop.”* (Participant 6).

#### 4.1.2 Showing empathy

For representatives of Generation Z, empathy is a significant tool that stands at the strategic point of communication. Generation Z, arguing that empathy should be the basis of communication, stated that it is not possible to establish correct communication without empathy while expressing that they will be tolerant towards their employees. Emphasizing the importance of understanding each other on the basis of communication in working life, the participants remark that peace in working life can only be achieved by means of communication and that empathy is the basis of communication.

*“Empathy is definitely needed. In other words, you cannot establish proper communication without empathizing with the person beneath you.”* (Participant 1).

*“I will definitely be empathetic. Because there will be people I manage, empathy is always necessary in this respect. I think the leader should have a lot of tolerance.”* (Participant 7).

#### 4.1.3 Egoless

Future leaders are disturbed by the presence of ego in the definition of the best leader. The important point to be emphasized in this sub-theme is that the presence of ego in teamwork is considered to be a negative characteristic among colleagues. Participants regard ego as a negative characteristic of their colleagues, but they also state that it is necessary to have a certain ego in order to climb the career ladder. Generation Z employees think that ego should decrease with time and experience, in that leaders consist of people who have developed themselves and are experienced.

*“In my opinion, a leader should be egoless, be able to manage people well in the place he works, and should not give room to ego in any way. After all, they are experienced people who have improved themselves in the field of business.”* (Participant 4).

*“The one who will pick me up when I fall is the one without ego, regardless of his position. This is the first thing. There should be no ego; ego has a very important place in the business field. If the leader wants to elevate himself/herself, of course he must have ego, but not against his teammates; his teammates will already elevate him. He is also the person who will elevate his teammates. At the end of the day, the appreciation is given to everyone. There should be ego in one’s own life, but not against your teammates.”* (Participant 6).

#### 4.1.4 Managing people well

For future leaders, management styles constitute a very important part of communication. In this sub-theme, the expressions that the participants focused on were about good management. Participants claimed that the way they use language is also effective in good management of leaders and stated that managers who use the “I” language are not liked, therefore “We” language should be preferred. Moreover, they consider that good management represents team spirit; therefore, it is essential that good management be objective and balanced.

*“While communicating with people in companies, especially senior managers, managers who say “I” are always more common. Such leaders may be disliked or misunderstood by their employees. Let us just do this instead. I think managers who talk like we can do it this way are always better. He needs to be able to direct and manage his team very accurately.”* (Participant 3).

*“The manager must be able to tell what is right and what is wrong objectively and manage his team accordingly.”* (Participant 10).

*“Being able to establish a balance between the top and bottom in the workplace is to ensure the workflow. Being able to manage the process well.”* (Participant 8).

#### 4.1.5 Managing with love and respect

Generation Z leaders have adopted the understanding of “love for the younger ones and respect for the elders,” which is a part of the Turkish culture, when it comes to managing representatives of different generations, younger and older than themselves, in the future. While they expressed that they would approach their younger employees with love when they became leaders, they also stated that they would communicate respectfully with their older employees and convey what needed to be done without offending them.

*“If he is older than me, I will manage him with a little more respect; if he is younger than me, I will manage him with love, but I still tell him that you have to do what is needed to be done accordingly. After all, if it is my job, I have to tell him that way.”* (Participant 5).

*“If someone from the older age group works for me and I am his leader, then my attitude towards him cannot be the same as the attitude I have towards someone 2 years older than me. You have to go to that side a little bit and not break them, you know, something is happening inside that side. “How many years he/she is younger than me is telling me what to do, so we should not let this be felt.”* (Participant 6).

### 4.2 Theme 2: working conditions

#### 4.2.1 Giving training

Education is a crucial tool for Generation Z. Based on this fact, future leaders have drawn attention to the importance of education in working conditions. Education is one of the most basic activities that will ensure development in working life. For this reason, the participants did not emphasize that the training should be face-to-face although they remarked that organizing training in the workplace is one of the effective tools to ensure the development of their teams. Digital natives, reflecting technological development in educational activities, express that regular in-house training should be organized in areas that individuals want and are deemed necessary.

*“I used to give constant training, and I am really happy that they are doing this in my company now.”* (Participant 13).

*“For example, it could be sending him to a course he wants, it could be meeting his needs, it could be online or it could be somewhere he can go; I attach great importance to this. I think I would be a developer and do something like this, for example, I would definitely do this.”* (Participant 12).

*“I am already working on this, there should definitely be regular in-house training, personal training and sports activities should be supported constantly.”* (Participant 8).

#### 4.2.2 Offering payment according to output

Wage is one of the basic outcomes of working life. Generation Z, who cares about offering equal payment for equal work, expresses in this sub-theme that wage levels should be determined according to output. Participant 5 emphasized the importance of the issue, suggesting that receiving the same wage for people doing different jobs in the same workplace lead to low motivation, which they experienced.

*“If everyone’s job is equal, I think of giving everyone equal salary, but if someone does more and works more, of course he deserves a higher salary.”* (Participant 11).

*“I think people who work in the same workplace but do different jobs should not be paid the same wages. Frankly, we experienced this situation, too. For example, I do more work than other employees, but I actually get the same salary as them. This is actually something that demoralizes me. That’s why, I pay attention to this issue and try to give him his due according to the work he does.”* (Participant 5).

#### 4.2.3 Preferring flexible working hours

Working hours in the IT sector are known to have flexible regulations. In this regard, all participants in the present study are people who have experienced flexible working. Despite this experience, they argue that working hours should be flexible. When it comes to hybrid work, which is the basis for determining working hours, it is expressed that it is necessary to be at work at the same time on certain days and hours, even it is distance working, and that it will be effective to assign the work for certain time intervals such as weekly, but it does not matter how many days or hours the employees accomplish these works. They think that employees should decide on this issue.

*“I think working hours should be flexible, but of course, there are certain hours of the day, when everyone should be in front of the computer for five or 6 h a day, but rather, it is not a matter of being in front of the computer, you can also work at eleven; and be in front of the computer between eleven and five so that you can work within the team. Let us finish our conversations, let us finish our agreements and work.”* (Participant 10).

*“I assign him a weekly job, for example, if that week’s job can be completed in 1 day, he can sleep for the other 4 days, as long as it is a job, and if he can earn the same money even if he does it in 5 days, if that job can earn the same money even if he does it in 1 day, this is his practical intelligence.”* (Participant 9).

#### 4.2.4 Being friendly

In this sub-theme, future leaders lay stress on the importance of a comfortable working environment. What is important for them is that there are no distances in the working environment and a friendly team is formed. Therefore, they want their teams to be friends with each other and with themselves. However, they also draw a line between friendship and emotional relationships.

*“I would like to have a team that is like a friend without distance, and I would like them to be like friends with me, as well and I would like the same to be experienced among them.”* (Participant 10).

*“Maybe I can call it emotional contact, not establishing emotional relationships, but being able to approach in a friendly manner.”* (Participant 8).

*“I can say that they prefer friendly, talkative leaders. Because Generation Z is a little more like this. Talkative person; let us be friends and hang out.”* (Participant 13).

#### 4.2.5 Assigning appropriate work to the employee

Assigning appropriate work to the employee not only ensures efficiency in working life, but also constitutes one of the crucial points in sharing job. Thus, participants think that one of the primary duties of leaders is to ensure effective management by observing who can do what job. Participants also point out that when they become leaders, they will assign appropriate work to employees.

*“So, I dream of being a leader in this regard, as I said, a leader who can organize people well and give people the jobs they can do.”* (Participant 4).

*“I definitely think I can usually organize what needs to be done or who needs to do what well.”* (Participant 7).

#### 4.2.6 Being fun

The working environment for Generation Z should be fun. Bearing this in mind, future leaders stated that they will make sure that the work environment is fun while organizing it. However, they will also pay attention to the fact that the work must be done properly while providing a fun environment. While Generation Z defines the fun they have with their colleagues, they give priority to celebrations such as happy moments, group events and social activities. Based on their own experiences, they also concentrated on the motivational side of these activities.

*“I think it would be a very fun environment if I were there. First of all, I think I would be a leader who avoids chaos but ensures that the job is done properly.”* (Participant 5).

*“Our leader once arranged a celebration. He had prepared a presentation or something for us. It was a small detail, saying “We have achieved these so far,” but it was still a motivating moment. There may be further unexpected events. When I think about it physically, happy moment can be a group event, like bowling. I would create such an environment.”* (Participant 1).

#### 4.2.7 Not giving importance to gender

Leadership is often attributed to men due to social gender. When we wanted to measure Generation Z’s perception regarding the gender of the leader, all participants constantly and repeatedly stated that the gender of the leader does not matter.

*“No, I do not have any problem with that issue; it can be a man or a woman.”* (Participant 3).

*“As a man, I do not attach much importance to it.”* (Participant 2).

*“It does not matter for me.”* (Participant 4).

All of the participants, who stated that the gender of the leader did not matter, thought of men as the first gender that came to their mind when the leader was mentioned. While the participants attributed the reasons for this to social gender roles, they also suggested that men are preferred more for this reason, and therefore, the number of male leaders is high in the sector and working life. However, while this situation increases the number of men, male leaders are encountered commonly as more opportunities are provided to men, which strengthens men’s position as leaders in society.

*“Men are thought to be more rational, and because women are socially assigned the role of motherhood, I think they become disqualified after a certain age. We can call it social gender; women are codes that have been developed for that.”* (Participant 8).

*“So when I think of a leader, the first gender that comes to my mind is male.”* (Participant 11).

*“So there is a situation like this; I do not know why it is preferred, although women can do the same things and men can do it equally, but the first reason which came to my mind is the leadership I see around me. Unfortunately, the first thing that comes to my mind is men, since men have mostly been leaders in very important positions in big and big places.”* (Participant 12).

## 5 Discussion

Due to the fact that leadership is a multi-dimensional and dynamic concept, it does not have a universal definition and is described by considering its different dimensions (Toma et al., 2020). Changing generations and their expectations in working life do not make it possible to make a single definition of leadership. Rather than limiting leadership to a uniform definition, one of the best ways to describe a good leader is that the leader knows and understands the expectations of his employees. How to be a leader? What are the behaviors that make a leader effective? Studies seeking answers to these questions are confronted frequently in the literature. In organizations, many leaders continue to behave inappropriately for leadership, and employees remain to be dissatisfied with their leaders (Koç et al., 2022).

Sustainability and efficiency of organizations are possible with satisfaction of the employees. Hence, we need to talk more about what kind of leaders Generation Z, who will take their place as leaders in the future, will be as well as knowing their expectations from their leaders. In the present study, an answer was sought to the question of what Generation Z’s perspective on leadership is and what kind of leaders they will become. The remarkable findings focused on two themes involving intra-organizational communication and working conditions.

The results indicate that Generation Z leaders will carry out their intra-organizational communication with the sub-themes of being supportive, empathetic, egoless, managing people well and managing with love and respect.

Being a supportive leader in the working environment means job satisfaction, productivity and high performance for employees (Kim et al., 2021). Incorporating employees into decision-making processes, resolving conflicts and maintaining the comfort of the working environment becomes possible if the leader is supportive in organizations (Dayanti et al., 2022). According to Generation Z, one of the significant qualities that an ideal leader should possess is that he supports, trusts and respects his employees (Aydoğdu and Kara Özkan, 2021; Gabrielova and Buchko, 2021). According to the results of the study, participants are realized to define providing support as one of the basic duties of a leader. They also attach importance to getting support from their teams while they clearly state that they will support their teams when they become leaders in the future.

Meanwhile, the ability to empathize, which is among the universal characteristics that a good leader should possess, improves commitment of employees to their organizations and their sense of trust and motivates Generation Z (Kasaroğlu, 2021; Gaan and Shin, 2022). From the perspective of Generation Z, a real and effective leader should show empathy, develop a friendly relationship with employees in the working environment and be humble (Singh and Dangmei, 2016; Grow and Yang, 2018; Aydoğdu and Kara Özkan, 2021; Diz, 2021; Dwidienawati et al., 2021; Demirbilek and Keser, 2022; Tidhar, 2022). The leader’s awareness of ego is important in that it contributes to the performance of employees in organizations and strengthens their psychological capital. A condition for creating this awareness is empathic communication. The leader’s humility affects the level of effective leadership (Poyraz and Atalay Cilveoğlu, 2020) positively, improves employees’ commitment to their organizations and their sense of trust, and motivates Generation Z (Kasaroğlu, 2021; Gaan and Shin, 2022). Similarly, for the participants, empathy is necessary for communication. According to findings in the present study, Generation Z attaches importance to empathy and being egoless in communication. Whereas they express that they will show empathy towards their own team, they are observed to be disturbed by the leader’s ego while working within the team. For this reason, they claim that they will leave the ego out of teamwork. Our findings coincide with studies on Generation Z’s expectations from leaders. It is thought that Generation Z will be empathetic and egoless leaders in the future when their expectations from leaders are considered.

Employees in current organizations consist of different generations. In this respect, a good leader should communicate within the organization within the framework of love and respect by taking these generational differences into account. The findings emphasize the significance of communicating with love and respect towards Generation Z employees, who will be the leaders of the future. Generation Z values leaders who have high-level of communication skills and provide guidance, and they also prefer a positive, sincere, love and respect environment in organizations (Nikolic, 2022). Every person desires to be cared for and respected, which also applies to employees. Employees strive for approval both from their peers and their leaders. If employees are appreciated and valued, they see themselves as a part of the team, which is reflected in the productivity of organizations (Notar et al., 2008). The results reveal that while the participants were discussing about good management, they laid stress on the fact that the leader should be balanced and objective especially in team management.

According to the results, it has been found that Generation Z leaders will determine the working conditions in their organizations with the sub-themes of giving training, offering payment according to output, preferring flexible working hours, being friendly, assigning appropriate work to the employee, being fun and not giving importance to gender.

It focuses especially on personal training in terms of managing and developing the Future Leaders team. While most of the participants supported technical training, they focused more on personal training. Future leaders argue that they will support their teams in training, especially to improve themselves, and that it does not matter whether the training is job-related or irrelevant as they state that the important thing is to get training in the areas they are interested in and really desire. In a study conducted with C-level managers (Klein, 2018), it is found that future employees’ graduation averages or certificates are not as important as experience; however, digital know-how, technological proclivity and on-the-job training are other important factors in addition to a good education. In accordance with the findings we obtained in the study, it is concluded that Generation Z leaders will organize in-service training.

One of the tools motivating Generation Z in their workplace preferences and expectations from business life is wage (Güleç Bekman and Gündüz, 2022). They are in favor of fair wages and transparency in payments (Urgal Saracho, 2023). The results showed that when the participants became leaders, they would pay equal wages for equal work and determine their wage levels according to output. Future leaders look more positively at flexible payment systems (Nieżurawska, 2023), which include impact-based wages, success-based payment, and skill/ability-based payment elements; and therefore, they do not prefer a single pricing system.

The IT industry concentrates on flexibility, adaptability and mobility. Those working in this sector can increase their performance in working environments when they can constantly learn, make their own decisions and encourage independence (Gaan and Shin, 2022). It is an expected situation for the IT sector that participants adopt flexible working hours when they become leaders. Generation Z leaders focus on getting the job done and emphasize that the employee determines working hours. In the study conducted by Tidhar (2022) with managers of high technology companies, it is stressed that managers need a new management style for Generation Z, which can adapt faster to the continuous development and change of technology by emphasizing the importance of flexibility and change while working with Generation Z. In this regard, the findings in the present study reveal that Generation Z will adopt flexible working hours when they become leaders.

For Generation Z, a friendly attitude is of great significance in effective leadership (Dwidienawati et al., 2021; Gabrielova and Buchko, 2021). At the same time, Generation Z attaches great importance to work and life balance and expect a friendly environment to be enabled for them at work. It is stated that providing this atmosphere will increase their loyalty to the company and they will adapt to changing conditions more easily (Bieleń and Kubiczek, 2020). In the study conducted to reveal the ideal manager profile of Generation Z, it is suggested by young people that being like a friend, who will protect and look after them, is important for the leader to be a reliable manager (Aydoğdu and Kara Özkan, 2021). Our research findings coincide with the literature. Future leaders want to be like friends with their teams. However, they think that this friendship should not turn into an emotional relationship.

Generation Z expects their leaders to act fairly. The sense of justice requires asking for the opinions of employees in the distribution of job descriptions according to clearly defined competencies of this generation, which will also ensure that the feeling of being valued, another expectation of Generation Z, is satisfied (Demirbilek and Keser, 2022). One of Generation Z’s expectations from working life is to work in companies that suit their skills and abilities and where they can improve themselves especially while looking for a job. They emerge as a generation admiring expertise and knowledge. They expect their leaders to give them jobs in accordance with their abilities in their career paths and consider this as an opportunity to improve their skills (Nabahani and Riyanto, 2020). Similar to the results in the present study, when Generation Z becomes a leader, it is realized that they will assign appropriate work to their employees and they are even observed to state that one of the main duties of leaders is to offer appropriate work to their employees.

Having a funny working environment for the participants is a motivating factor for them. It is proved in many studies that a fun working environment increases motivation, team culture and productivity (Pekdemir et al., 2018). A fun attitude at work increases employees’ commitment to the organization and their colleagues and also influences their intention to leave the job (Bilginoğlu and Yozgat, 2018). Generation Z leaders think that a working environment that is fun and organizes fun events will benefit both themselves and their employees. In this regard, it is observed that they are willing to plan collective events.

While leadership is attributed to men in societies, characteristics such as ambition, assertiveness and being aggressive are regarded as a necessity of being a leader and are considered masculine characteristics (Bornman, 2019). All of the participants, regardless of gender, thought of men when they mentioned leaders. However, the main reason is realized to be related to the leaders they see around them rather than their leader characteristics. While all of the participants stated constantly and repeatedly that the gender of the leader did not matter, they associated the fact that the men first came to their mind with social gender.

The present study aimed to determine what characteristics Generation Z will have when they become leaders by evaluating Generation Z’s view of leadership. The findings we obtained from the participants indicate that digital natives, the leaders of the future, expressed their leadership characteristics under two main themes which involve intra-organizational communication and working conditions. This determination constitutes an important finding of the study.

## 6 Conclusion

It is obvious that Generation Z, which has taken its place in today’s organizations, will assume leadership roles in the near future. In this respect, a good and effective leader should have characteristics such as being able to empathize with Generation Z, managing in a friendly manner with love and respect, paying wages according to output and preferring flexible working hours. The perspective of Generation Z on leadership and the type of leader they will become when assuming leadership roles, in a time where their presence in the workforce is expected to increase even further, is of great importance for organizations. The perspective of Generation Z, whose number of employees is expected to increase in the near future, on leadership and what kind of a leader they will be when they take on a leadership role is of great importance for organizations.

In this study, semi-structured interviews were held with 13 Generation Z participants who were employed in the IT sector. Two themes were identified in the analysis of the data obtained as a result of these interviews, which involve communication and working conditions within the organization. In the findings obtained from the interviews, it is realized that generation Z will determine how to carry out intra-organizational communication in organizations with the sub-themes of supportive, showing empathy, egoless, managing people well, managing with love and respect while they will determine the working conditions in organizations with sub-themes such as giving training, offering payment according to output, preferring flexible working hours, being friendly, assigning appropriate work to the employee, being fun and not giving importance to gender. In the light of these findings, it has been concluded that Generation Z leaders will fulfill these roles with the leadership characteristics mentioned when they assume a leadership role. However, it has been observed that Generation Z will have people-oriented and people-valuing leadership characteristics when they take on a leadership role.

Currently, changes in working life are much faster and more advanced than in the past. It is much easier for Generation Z to keep up with the pace of changing technology due to the fact that they can follow and adapt to changes in technology faster than previous generations. These features also affect their characteristic structure and they show these features in their working life. A good leader must understand the team he manages and know their expectations. Leadership is the art of managing people. It is important for leaders to know the expectations of Generation Z. Furthermore, it is crucial that they are cared for. When Generation Z becomes a leader, they want to implement a team management caring about employees and meeting their expectations. Bearing this in mind, organizations need to invest both in today’s leaders and Generation Z, who will be in leadership positions in the future. Additionally, Generation Z is recommended to be provided with training opportunities that will develop and strengthen their leadership skills with development opportunities within the organization as these investments will help employees realize their career plans, increase the efficiency of organizations and most importantly will affect employee satisfaction with the value given to people. More studies are needed to be conducted in different sectors to understand what kind of leaders Generation Z will be and their leadership skills. This study may shed light on future studies on what kind of leaders Generation Z will be.

## Data availability statement

The original contributions presented in the study are included in the article/supplementary material, further inquiries can be directed to the corresponding author.

## Ethics statement

The studies involving humans were approved by Trakya University Social and Human Sciences Research Ethics Committee Decision no: 2023.08.18. The studies were conducted in accordance with the local legislation and institutional requirements. The participants provided their written informed consent to participate in this study.

## Author contributions

BY: Data curation, Writing – review & editing, Writing – original draft, Investigation, Formal analysis, Conceptualization. EDK: Data curation, Writing – review & editing, Writing – original draft, Methodology, Formal analysis. SGK: Visualization, Writing – review & editing.
